# DEVELOPMENT OF A PHYSICAL PERFORMANCE SUMMARY SCORE IN MIDDLE AGE: FINDINGS FROM THE CARDIA FUNCTION ANCILLARY STUDY

**DOI:** 10.1093/geroni/igad104.3273

**Published:** 2023-12-21

**Authors:** Brett Burrows, Richard Sloane, Carl Pieper, Cora Lewis, Kelley Pettee Gabriel, David Jacobs, C Barrett Bowling

**Affiliations:** Duke University School of Medicine, Durham, North Carolina, United States; Duke University Medical Center, Durham, North Carolina, United States; Duke University School of Medicine, Durham, North Carolina, United States; University of Alabama at Birmingham, Birmingham, Alabama, United States; University of Alabama at Birmingham, Birmingham, Alabama, United States; University of Minnesota, Minneapolis, Minnesota, United States; Duke University, Durham, North Carolina, United States

## Abstract

Recent evidence suggests that ~20% of middle-aged adults report mobility limitations, supporting the need for assessment strategies to detect early-onset limitations, appropriate for this population. Coronary Artery Risk Development in Young Adults (CARDIA) enrolled 5,115 Black and White men and women ages 18-30 years from four sites (Birmingham, AL; Oakland, CA; Minneapolis, MN; and Chicago, IL) in 1985-86. The CARDIA Function Study added self-reported function (PROMIS SF20a) and 5 physical performance measures (gait speed, single-leg balance, lower extremity strength, grip strength, and 6-minute walk test) to the Year 35 in-person follow-up exam conducted in 2020-22. The CARDIA Physical Performance (CAPP) score was developed by calculating sex-specific quartiles separately for each performance measure, assigning points based on these quartiles (0 for not attempted, 1-4 for each quartile), and summing points across all 5 performance measures (range 0-20, higher scores = greater performance). Among 2,021 CARDIA Function participants [mean age: 60.1 (±3.6) years; 57% female; 45% Black] the mean CAPP score was 12.3 (±4.1; range 0-20). Mean CAPP scores differed by age < 60 vs. ≥60 years old (12.7 vs.12.1, p< 0.05) and self-reported function above vs. below the median SF20a score (14.0 vs. 10.6, p< 0.001), but as expected, not sex (12.3 vs. 12.3, p=0.99). Among middle-aged adults, the newly developed CAPP score captured a wide range of physical performance. Lower CAPP score was associated with lower than median self-reported function suggesting that the CAPP score may be helpful assessment tool for detecting early-onset limitations to attenuate functional declines with aging.

